# COVID-19 and a novel initiative to improve safety by 3D printing personal protective equipment parts from computed tomography

**DOI:** 10.1186/s41205-020-00073-6

**Published:** 2020-08-12

**Authors:** John J. Coté, John Haggstrom, Ranuga Vivekanandan, Kristin A. Coté, Daniel L. Real, David P. Weber, Anne Cheng, Nicholas G. Dubay, Robin Farias-Eisner

**Affiliations:** 1 Department of Obstetrics and Gynecology CHI Health, Creighton University School of Medicine, Omaha, NE USA; 2 Department of Radiology, Creighton University School of Medicine, Omaha, NE USA; 3 Department of Medicine Division of Infectious Disease CHI Health, Creighton University School of Medicine, Omaha, NE USA; 4 Creighton University School of Law, Omaha, NE USA; 5 Creighton University School of Medicine, Omaha, NE USA

**Keywords:** COVID-19, Computed tomography, 3D printing, Personal protective equipment, Powered air-purifying respirator

## Abstract

**Background:**

Powered air-purifying respirators are in short supply and can break down with extended use. Replacement parts can become hard to acquire. The aim of this study was to create an innovative quality improvement proof of concept using rapid prototyping.

**Methods:**

Here we report three cases of 3D printed powered air-purifying respirator parts. 3D printing was performed on all parts using fused deposition modeling with standard polylactic acid, in the same way that presurgical models would be created. Measurements using an electronic caliper as well as CT scans were used to compare an original part to its corresponding 3D printed parts for accuracy.

**Results:**

Electronic caliper and computed tomography measurements both showed accuracy consistant with current published norms.

**Conclusions:**

Ultimately, there will be questions surrounding intellectual property, effectiveness and potential long-term safety for these types of 3D printed parts. Future research should look into the addition of specific nanoparticles from the position of cost, efficacy, safety and improved accuracy.

## Background

The COVID-19 pandemic has been pushing hospital systems and caregivers to the brink [1, 2]. One of the many areas that have been critical in the fight against the novel corona virus is personal protective equipment (PPE). The fact that this respiratory virus is known to spread via droplets and possibly smaller aerosolized particles means some of the most important protective equipment are N95 masks and powered air-purifying respirators (PAPR) [3–6]. At the time of this case series the Centers for Disease Control and Prevention (CDC) in the United States has recommended N95 or higher level respirators for all aerosol generating procedures [7, 8]. There are potential advantages to using a PAPR over the N95 respirator; PAPR devices are more comfortable, limit inadvertent facial touching, avoid issues of compromised fit, improve efficiency, are more reusable, and have been shown to be more effective at protecting healthcare workers [9–15]. Some of the disadvantages including cost, loss of visual acuity, and being noisy, have encouraged stakeholders to recommend a combined approach in using both N95 respirators and PAPR devices [16]. Regardless of the pathway individual hospital systems around the world have chosen, a critical shortage of these particular PPEs is having a detrimental effect on caregivers and the patients they are trying to help. Due to this urgent need, many have explored inventive options to create solutions for the shortage of PPE and replacement parts [1, 6, 9]. Even with encouraging news on sterilization and reuse of N95 respirators, [17] 3D printing has been at the forefront of technological solutions during this unprecedented pandemic [1, 2, 18]. This collaborative project was undertaken as an essential quality improvement innovation initiative in the setting of the COVID-19 pandemic.

## Methods

At our institution the increased use of N95 respirators and PAPRs for PPE has produced unique issues due to the enormity of the pandemic and the sheer quantity of needed units. Three different PAPR units are available in Catholic Health Initiative (CHI) hospitals. All units include a headpiece and a reusable blower unit with filter, battery, and hose. With the increased use of the PAPRs, the breathing hose on the units is one component that was in need of repairs. The Air-Mate™ PAPR was the one most commonly used within our institution. The feasibility of an innovative idea or concept to solve a problem is by definition what a proof of concept entails. This proof of concept innovative quality improvement project was therefore evaluated by the ability to produce a workable replacement part, specifically the Air-Mate™ PAPR. We accessed the accuracy of the 3D printed parts in two ways. First, we selected five regions (large opening external diameter, small opening external diameter, height, large opening internal diameter and small opening internal diameter) on one of the Air-Mate™ PAPR ends. Measurements were performed with an electronic caliper to the nearest 0.01 mm. Three 3D printed replacement parts were measured at the same regions. Each region was measured twice. Second, we performed a CT scan of the original PAPR part and the same three 3D printed replacement parts. We measured the external diameter of the small opening and the large opening. Diameter measurements were performed twice perpendicular to each other. Measurements were done to the nearest 0.1 mm.

Statistical analyses were conducted using descriptive statistics (means and standard deviation) considering *p* ≤ 0.05. Dimensional error was calculated as the absolute difference (mm) between the values obtained from the 3D printed part and those from the original PAPR part. Relative differences (%) were calculated as the absolute difference divided by the original PAPR part value multiplied by 100 as referenced by previous studies [19, 20].

## Interventions

### 3 M™ (St. Paul, MN USA) air-mate™

**Fig. 1:**
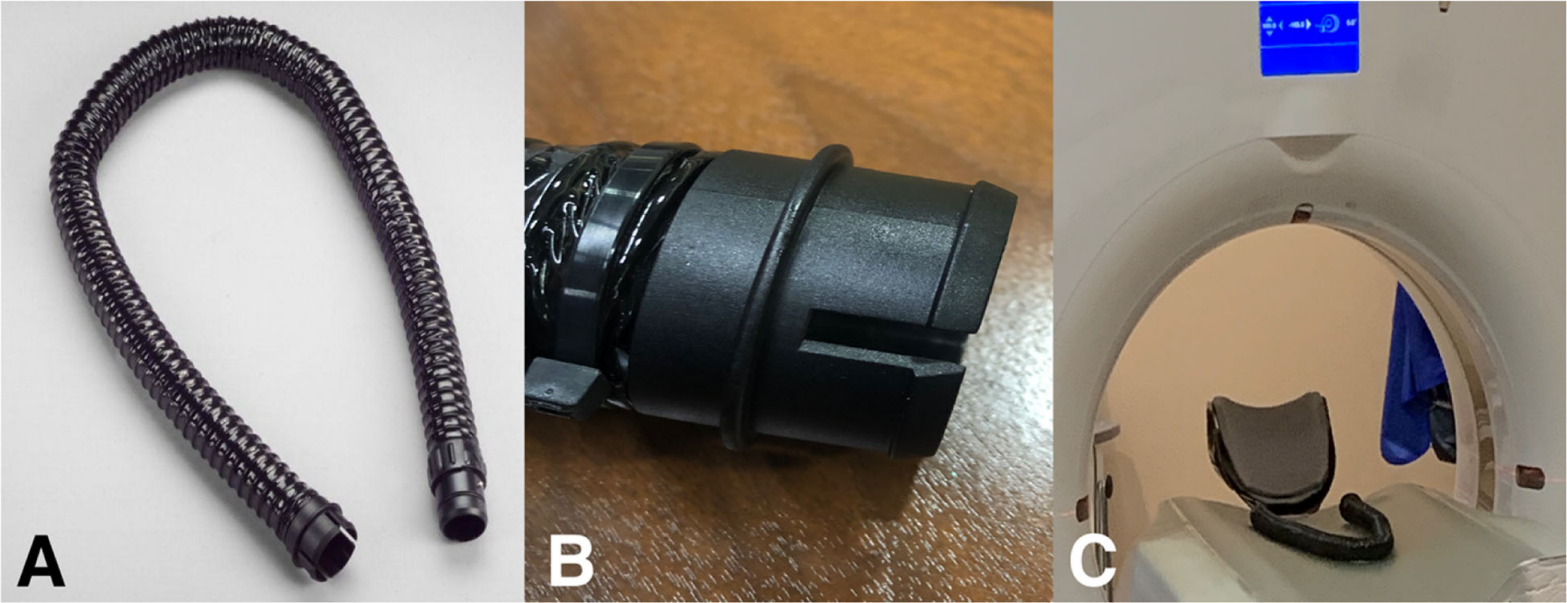
**a** 3 M™ Air-Mate™ breathing tube. **b** Hose attachment that is often the failure point on the tube. **c** Tube being scanned in Siemens™ SOMATOM Definition Edge™

**Fig. 2:**
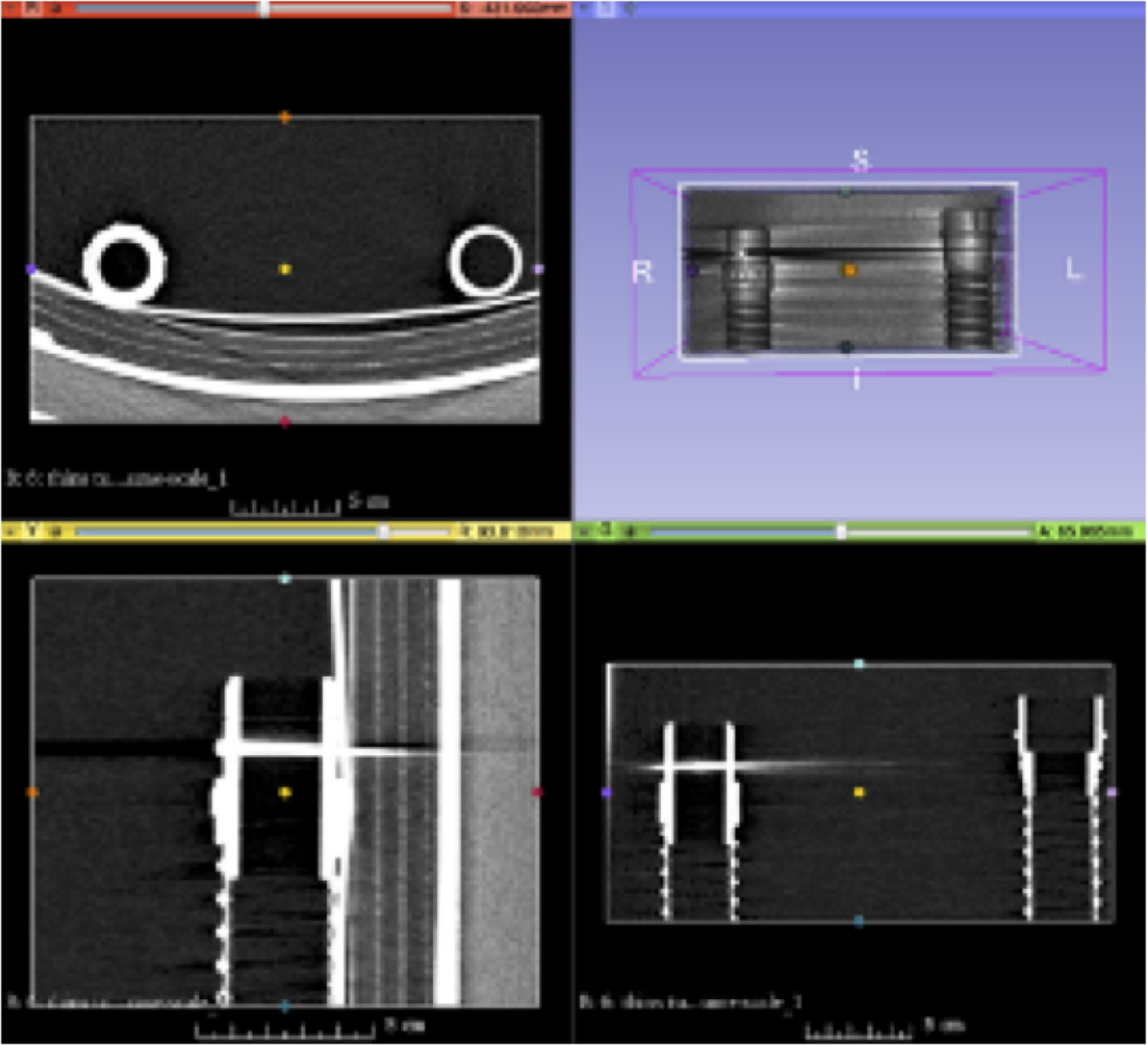
3D Slicer software used for image processing and three-dimensional segmentation

**Fig. 3:**
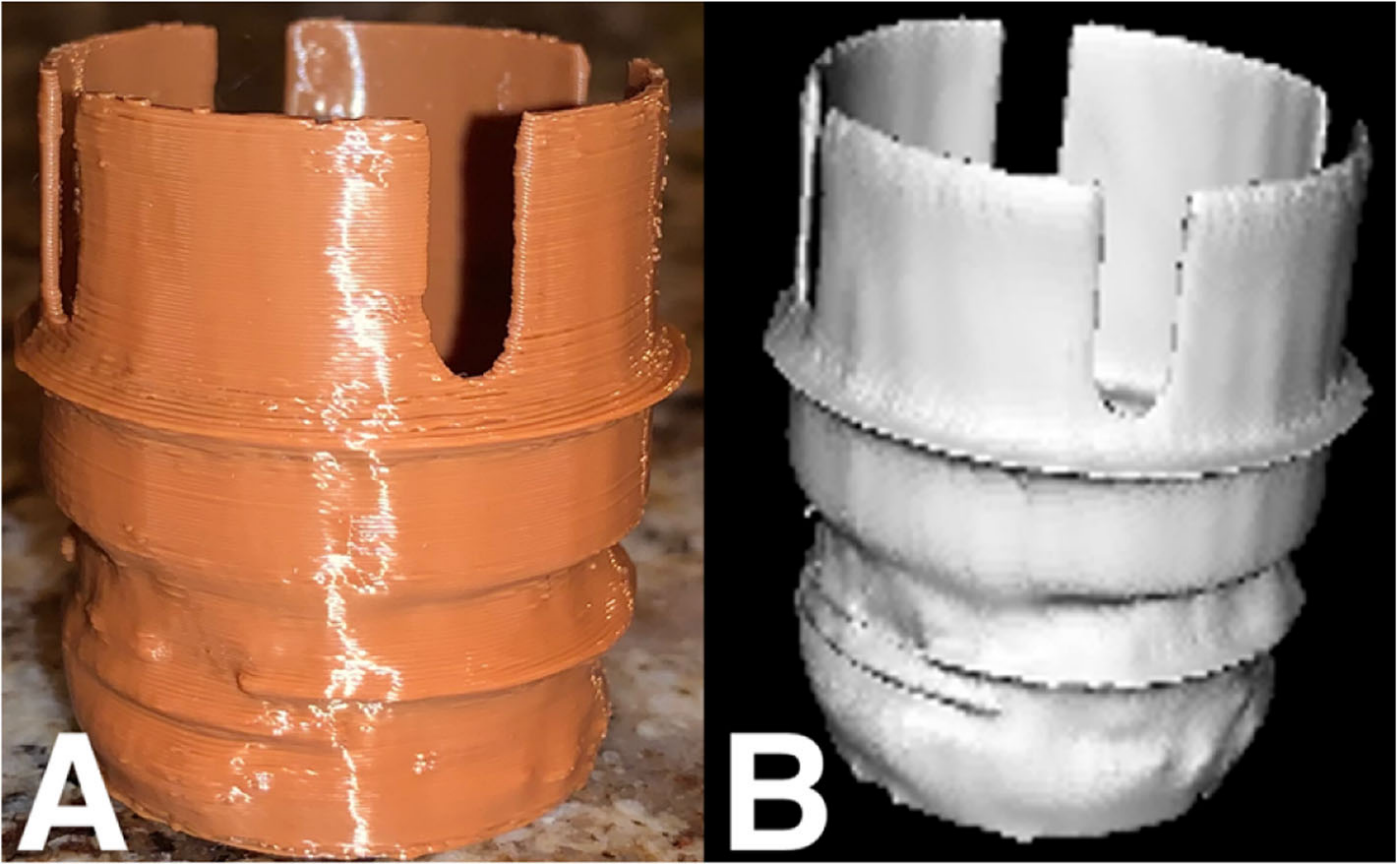
**a** 3D Printed, final product with hose attached. **b** STL file of replacement tube attachment

**Fig. 4:**
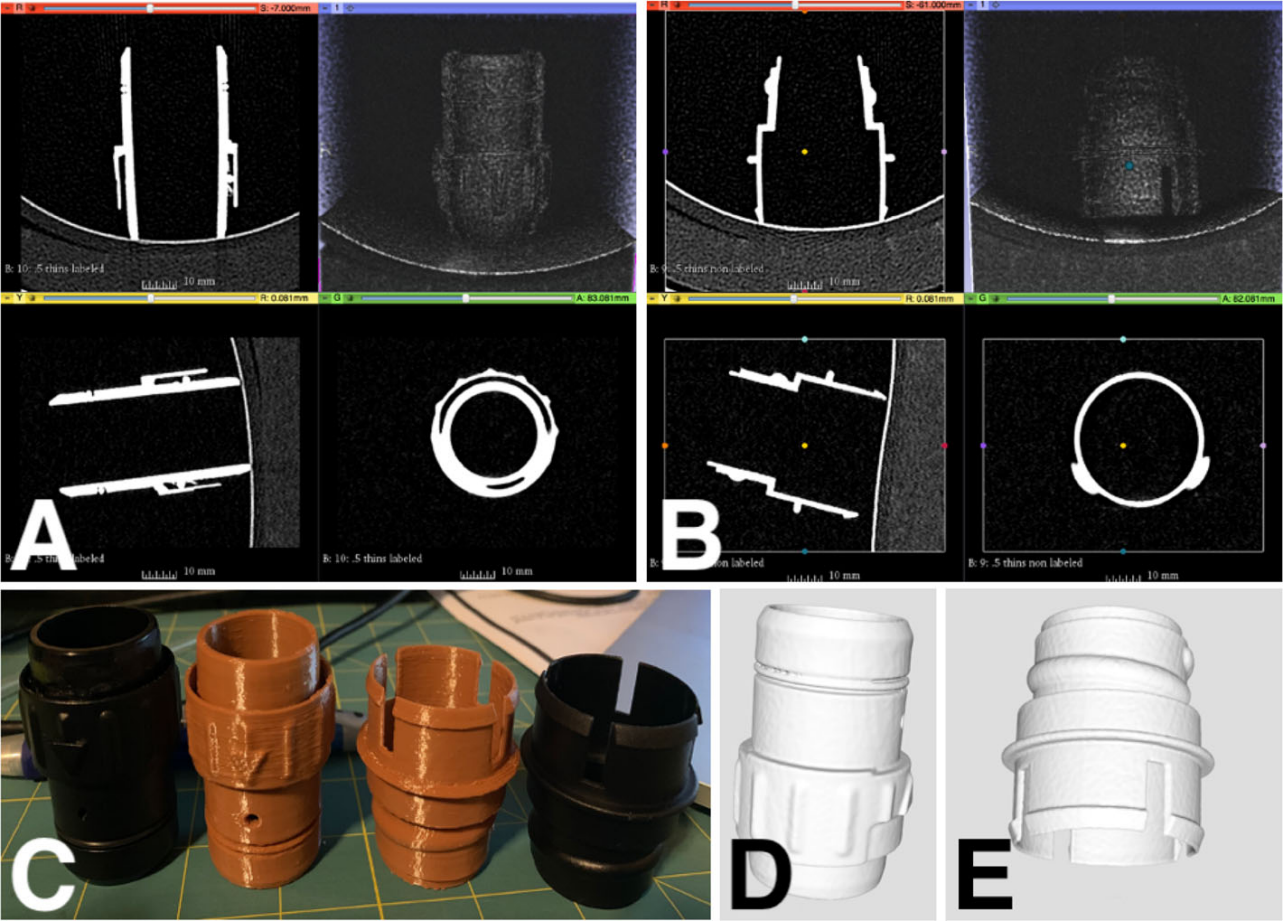
**a** & **b** 3D Slicer image processing and segmentation seen in x, y, and z planes as well as 3D render. **c** Side-by-side comparison of original vs. 3D printed parts after hoses removed. **d** & **e** STL files of 3D parts

This battery-powered air purifying respirator features an all-in-one design, which draws air through a filter or cartridge to provide respiratory protection to the wearer. The manufacturer has discontinued this particular unit, making the breathing tube replacement parts unavailable and difficult to find. There have been numerous good faith attempts to procure replacement breathing tubes with poor results. Consequently, alternative options have been required, including attempting to replicate broken parts using additive manufacturing. Initially the tube ends were scanned on a Siemens™ (Munich Germany) SOMATOM Definition Edge™ CT scanner (Fig. 1). A total of 611 slices were performed at 0.5 mm thickness and reconstructed at a 0.2 mm interval. Reconstruction field of view was 120 mm. Voxel size was 0.23 × 0.2 × 0.2 mm. A tube current of 120 kV with a reference mAs of 180 was utilized. A reconstruction kernel of Hr40s, with sharp edge enhancement was performed which is typically utilized with sinus CT imaging. The digital imaging and communications in medicine (DICOM) data was exported to a CD ROM and was reconstructed in 3D Slicer (the open source software platform for medical image informatics, image processing, and three-dimensional visualization) for segmentation [21–24]. (Fig. 2) After converting the model to a stereolithography (STL) file, the file was exported to the Cura Lulzbot Edition©(Fargo, ND USA) v3.6.20 to create the g-code. The g-code was exported to a Lulzbot® (Fargo, ND USA) TAZ Workhorse 3D printer and the parts were printed with polylactic acid (PLA). (Fig. 3) A 4 mm nozzle with the following printer settings was used: Layer height .09 mm, wall thickness .08 mm, bottom thickness .5 mm infill density 18%, print speed 60 mm/s, infill speed 60 mm/s, outer wall speed 50 mm/s, inner wall speed 50 mm/s, travel speed 150 mm/s, retraction enabled, and the heated bed was set to 50 degrees Celsius. After imaging each connecter from the hose separately, the process was repeated, and the parts were again 3D printed (Fig. 4).

### ILC Dover (Frederica, DE USA) sentinel XL™

**Fig. 5:**
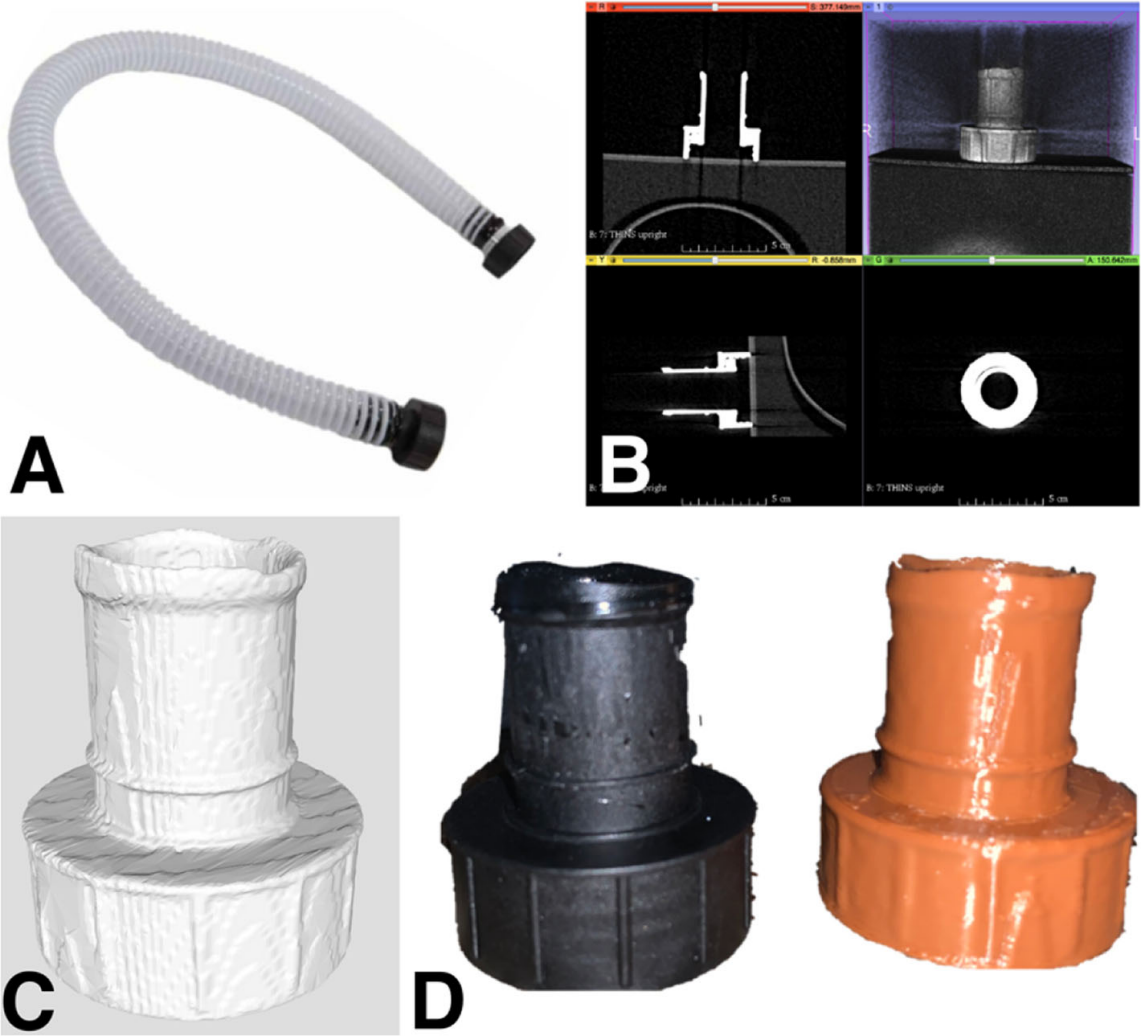
**a** ILC Dover Sentinel XL™ hose. **b** 3D slicer image processing and segmentation. **c** STL of hose connector. **d** 3D printed hose connector

This unit is similar to the Air-mate™ but the breathing hose connections are not interchangeable with the other models we possess. This unit needs a threaded breathing tube connection. Although this model was not discontinued, ordering new units or even replacement parts would not meet the current demand, and again alternative options were required. Both ends of the breathing tube are the same and one end was scanned without the breathing hose attached, it was segmented in 3D Slicer, converted to an STL file and printed with the same settings as the Air-Mate™ (Fig. 5).

### 3 M™ (St. Paul, MN USA) Versaflo™

**Fig. 6:**
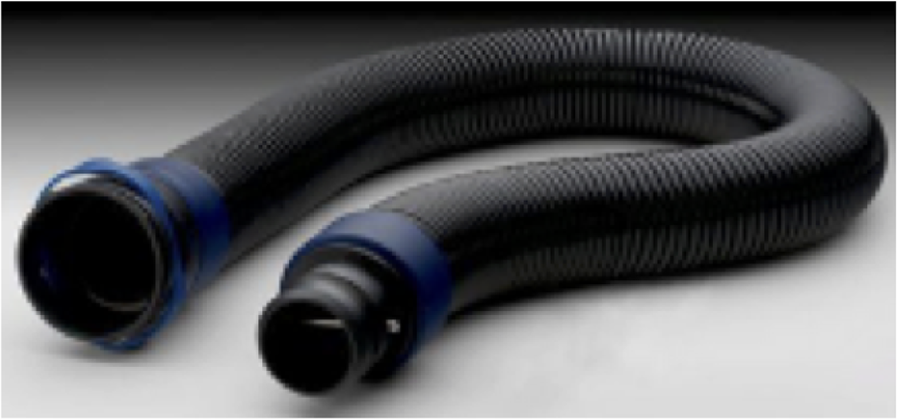
3 M™ Versaflo™ breathing tube

**Fig. 7:**
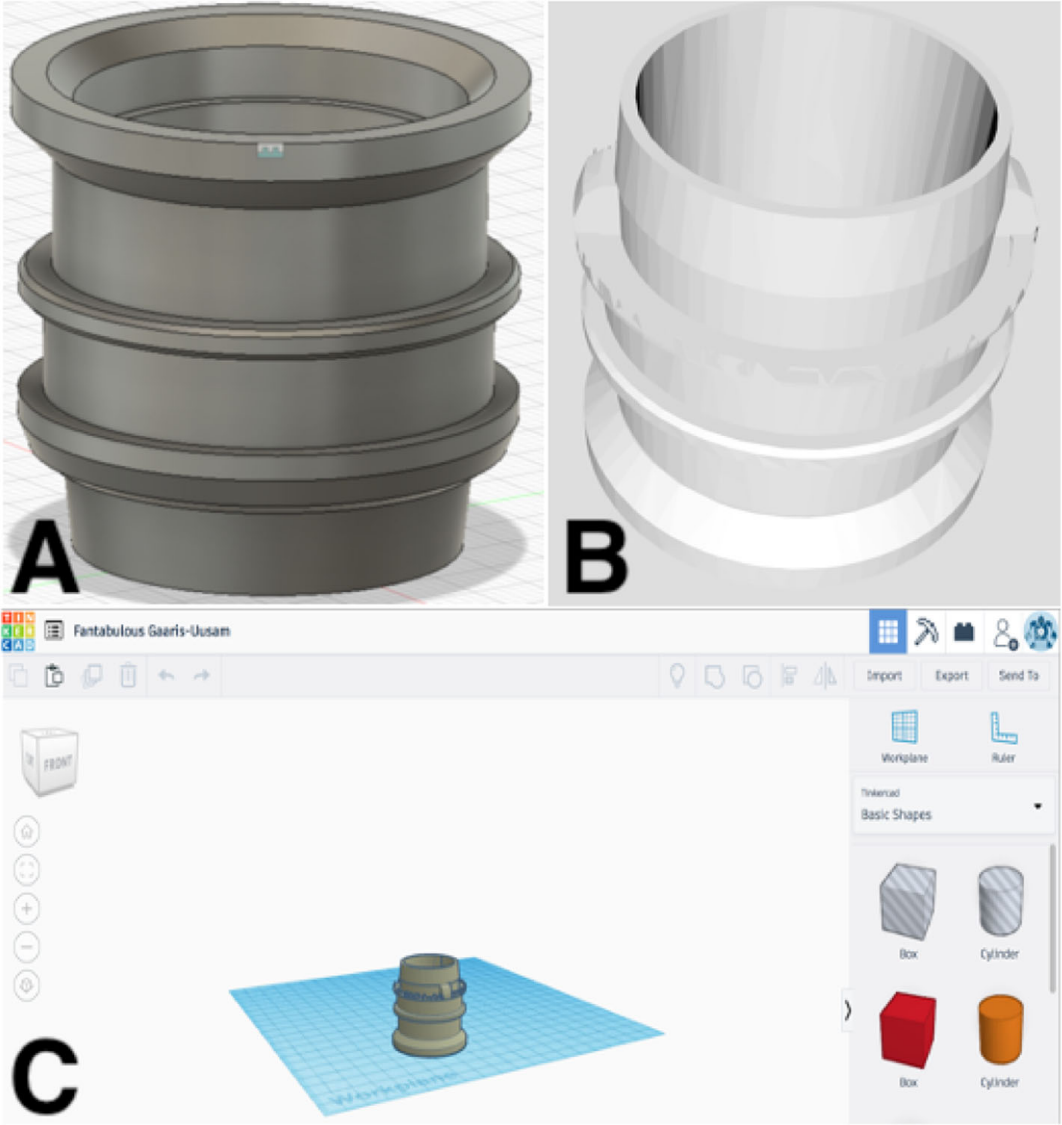
**a** Generic STL file of PAPR hose connector. **b** Final STL file of hose connector ready to be printed. **c** Altered version of hose connector in tinkercad™

This PAPR system is for particulate matter. This model has gone through a few upgrades. As with most technology, individual parts can be unique to a company or even to a particular version. Unfortunately, this breathing tube connection was not usable in the other versions of 3 M PAPRs and vise-versa. (Fig. 6) This particular PAPR was not used at our institution on a regular basis so instead of creating a replacement part with CT scanning we tried to find an open source solution. An open source STL file [25] was downloaded that was created to fit generic PAPR hoses and was tested on the 3 M™(St. Paul, MN USA) Adflo™ PAPR System. Alterations were needed for this 3D printed part to fit the Versiflo PAPR. The STL file was altered in tinkercad™ (San Francisco, CA USA) to be able to fit the PAPR breathing hose. (Fig. 7) As previously noted, we used the same printer settings.

## Results

Mean absolute and relative differences of replacement parts compared to an original part is shown in Table 1. The results show that CT dimensions of three 3D printed parts (dimensional error = 0.72%) compared to CT dimensions of the original part on which they were produced was well within previously published norms [19, 20]. Mean absolute and relative differences of replacement part measurements by an electronic caliper are seen in Table 1. The total mean relative difference (1.73%, 95% CI [1.09, 2.37]) and total mean absolute difference (mean = 0.64 mm, 95% CI [0.39, 0.89]) are also listed.

**Table 1:** Mean absolute (mm) and relative (%) differences for 3D printed parts in comparison with the original PAPR

	Absolute CT (mm)	Absolute caliper (mm)	Relative CT (%)	Relative caliper (%)
Part 1				
Ext. Large	0.15	0.58	.39	−1.89
Ext. Small	0.45	0.15	.82	−0.47
Int. Large		1.44		−4.01
Int. Small		0.135		−0.49
Height		0.99		2.09
Part 2				
Ext. Large	0.35	0.58	0.9	−1.52
Ext. Small	0.2	0.1	0.65	−0.31
Int. Large		1.545		−4.36
Int. Small		0.37		−1.33
Height		1.06		2.24
Part 3				
Ext. Large	0.1	0.425	0.26	−1.11
Ext. Small	0.4	0.005	1.31	−0.02
Int. Large		0.96		−2.67
Int. Small		0.38		−1.37
Height		1.005		2.12
Total mean Relative Difference	0.275	0.64	0.72	1.73

## Discussion

Caliper measurements and CT measurements objectively confirmed, and our frontline providers subjectively agreed that the replacement parts were equivalent. Errors are inevitable within any measurement construct [19], yet in terms of the level of accuracy needed when 3D printing these replacement parts we are in uncharted territory. The precision required to support the acquisition, production and utilization of these replacement parts have not been established. Without any industry standards or regulations and in the current pandemic environment the minimum requirement for accuracy with 3D printed replacement parts should be a correct fitment with no leakage. The medical applications of 3D acquisition and 3D printing are described by some as transformative. There are tremendous advantages in the 3D printing space where reconstructed models using 3D rapid prototyping allow replication of sophisticated anatomical structures that can be used to facilitate anatomic study, surgical planning, and device development [26–31]. Additionally, 3D printing of 3D ultrasounds has also been recently shown to improve maternal-fetal attachment [32]. During this unprecedented time where 3D printing or additive manufacturing are producing unique 3D devices to mitigate COVID-19, [18] the ability to combine medical computed tomography (CT) with industrial metorology and CT is paramount. The industrial purpose of CT is much like the medical purpose, to image the internal and external areas of an object/person [33, 34]. The accuracy of 3D models is affected by errors at each step of the process, from the imaging of the components to the final printed product. Studies have shown relative accuracies of 3D printing on consumer printers of 2.2% +/− 1.8 [35]. The connecting material was ideal for CT imaging given the high contrast and absent artifact. The spatial resolution of the images is near the lowest feasible level, near 0.1–0.2 mm, in the x-y dimension for most 3D printers [25]. While the optimal amount of PPE supplied by device manufacturers would be ideal, the pandemic has made caregivers, hospitals and countries react quickly to protect ourselves and our patients through innovative solutions. In the three previous cases discussed above, there was a critical need to replace a piece of a PAPR under time sensitive conditions. In each instance, 3D printing was used to temporarily reproduce a part needed to fix a vital component of one of the most important protections to help fight the COVID-19 pandemic. While the proof of concept has been shown as potentially viable in the unprecedented setting of a global pandemic, there are a few weaknesses that need to be addressed.

## Patent issues

The issue of intellectual property being at odds with this unprecedented global pandemic needs to be examined. A patentable invention grants its inventor certain exclusive rights and a process patent protects the method by which the product is made. A recent case in Italy made worldwide headlines when a hospital and team of engineers designed and printed a digital version of a replacement ventilator valve to combat shortages occasioned by the COVID-19 pandemic [36]. The Open COVID Pledge asks intellectual property (IP) owners to voluntarily forgo asserting IP violations during the crisis, and to wait for one year after the World Health Organization (WHO) declares the pandemic to be over before asserting intellectual property right violation claims [37]. However, that pledge requires voluntary adherence by the patent holders. More directly, the U.S. Department of Health and Human Services (DHHS) is conferring tort immunity pursuant to 42 U.S.C. §247d-6d (the Public Readiness & Emergency Preparedness “PREP” Act) and 21 U.S.C. §§564A-B (the Pandemic and All-Hazards Preparedness Reauthorization Act or “PAHPRA”). As patents and copyright infringement claims are generally considered to be tort claims and fall under U.S. federal law, it appears as though health care providers acquiring the information needed to replicate the necessary parts of the breathing tube via CT scan, at this time, would be protected from copyright and patent liability [38–40]. The PREP ACT and PAHPRA are both limited in scope as to who qualifies for immunity. Only individuals and entities who meet the definition of “Covered Persons” who are engaged in the “manufacture, distribution, administration, or use of medical countermeasures,” or of “qualified pandemic and epidemic products” will receive liability immunity through Oct. 1, 2024 [38].

## Techniques

We only produced the parts with fused deposition modeling (FDM). We looked at the (FDM) method of additive manufacturing due to the ease, low cost, and ubiquity of this 3D printing technique. 3D printing a replacement part typically involves four steps: imaging, segmentation, slicing, and printing. Imaging is the process of acquiring a DICOM file via CT, magnetic resonance imaging (MRI), positron emission tomography (PET), or ultrasound scans. CT scans are usually the choice of imaging modality to pair with 3D prints. The DICOM file can be visualized, trimmed, and converted into a stereolithography file (STL) through the segmentation process. The STL file can then be prepared for printing. The American Society for Testing and Materials (ASTM) identifies seven broad methods for additive manufacturing (binder jetting, directed energy deposition, material extrusion, material jetting, powder bed fusion, sheet lamination, and vat photopolymerization), [41] yet only one method, FDM, was used to produce the replacement parts in the three cases. FDM 3D printing adheres melted thermoplastic in subsequent layers until the desired shape is formed. Most commercial 3D printers have the ability to print 30 μm between layers in theory. The COVID-19 virus has a diameter of approximately 60–140 nm [42]. Although not approved by the U.S. Food and Drug Administration (FDA) and not studied in a randomized clinical trial, some recommend a minimum wall thickness of 1.7 mm when printing masks, and have suggested altering slicer settings over the extruder [43]. These minimum recommendations point to the fact that there are more mechanisms involved, such as electrostatic charge, that stop the COVID-19 virus from penetrating manufactured or 3D printed N95 masks. We did not compare the different methods of 3D printing but PolyJet and resin printers can achieve less gaps between layers compared to FDM printers and may be better equipped to print N95 masks or replacement PAPR parts. Several factors must be taken into consideration to ensure safety, replicability and cost effectiveness of 3D printed parts. While a significant amount of research needs to be done before advising any particular process there is some evidence that 3D printing via FDM may help during emergency situations.

## 3D-filaments

We only used one type of polymer, PLA. The FDA has issued an Emergency Use Authorization (EUA) for medical devices (under section 564 of the Federal Food, Drug, and Cosmetic Act) including NIOSH-Approved Air Purifying Respirators, but has not commented on the individual parts used in these PAPRs [44]. The FDA evaluates and may approve a material as part of the finished device and its intended use, it does not evaluate the material itself. A variety of FDM filaments exist that have been FDA approved within medical devices, and potentially could be used to print these parts; PLA, thermoplastic elastomer (TPE), thermoplastic polyurethane (TPU), polycaprolactone (PCL), nylon, polyethylene terephthalate (PET), polyethylene terephthalate glycol-modified (PETG), polyethylene cotrimethylene terephthalate (PETT), and polyether ether ketone (PEEK). The FDA does list a variety of food safe materials (filaments) in the code of federal regulations [45]. These polymers have been approved as an article or component of articles intended for use with all foods under certain conditions; acrylonitrile butadiene styrene (ABS), polycarbonate (PC), polyvinyl alcohol (PVA), high impact polystyrene (HIPS), polyoxymethylene or acetel (POM), polymethyl methacrylate or acrylic (PMMA), flexible polyester (FPE), high-density polyethylene (HDPE), thermoplastic copolyester (TPC), acrylonitrile styrene acrylate (ASA), polypropylene (PP), and polyphenylsulfone (PPSU). While materials need to be biocompatible, inert, durable, and easily moldable, in relation to implants for patients, [46] these traits are also important for 3D printed PPE. When choosing the type of filament, material properties such as mechanical strength, elasticity, and the ability to sterilize must be considered in conjunction with end design and functionality. With additive manufacturing there are a multitude of materials to print with, but only one material, PLA, was used to produce the replacement parts in the cases described above. Two of the most common filaments used in healthcare are PLA and acrylonitrile butadiene styrene (ABS). Where PLA is made from starch and is biodegradable with moisture at 140 degrees Fahrenheit, ABS is made from petroleum and is not biodegradable. PLA is an inexpensive, and versatile material that can be sterilized and modified in several ways. PLA sterilization can be done with hydrogen peroxide, ethylene oxide, gamma irradiation, and electron beam with minimal change in its mechanical properties [47]. Additionally, postprocessing techniques, such as iodine coating and side chain modification for hydrophilicity, can further enhance antibacterial properties [48]. Although PLA has promising potential, its use for direct body contact has not been approved by ISO 10993 because of its incompatibility with high temperature sterilization techniques [49]. However, alternative sterilization options exist, and PLA’s non-cytotoxic and biodegradable qualities make it desirable for use during the COVID-19 pandemic [48]. PLA could be a good choice of filament to use in a PAPR, yet one caveat is that PLA absorbs moisture over time and can potentially affect mechanical integrity of the print [17, 50].

## Future options

PPE needs to protect both the patient and the caregiver. The primary mechanism for this is the barrier they produce. Due to COVID-19, reuse and sterilization have been examined to extend the life of scarce N95 masks and PAPRs [17]. A secondary mechanism to help prevent the spread of COVID-19 may include imbedding material within a filament or resin to improve the antimicrobial activity of the 3D printed object. With our study, the limitations related to sterilizing FDM 3D printed PAPR replacement parts may be decreased if the right material could be polymerized within the thermoplastic. There are continual advances in combining other materials to PLA to improve its antimicrobial activity. Sandler et.al. impregnated the antibiotic nitrofurantoin within PLA [51]. While there are commercially available filaments that include copper, there is ongoing research into additional materials that can be used to improve the antimicrobial nature of 3D printed devises; silver, [52, 53] MgO, ZnO and TiO_2_ [54].

## Titanium

Titanium nanoparticles have been shown to be a useful antimicrobial [55, 56] against bacteria. Additionally, titanium oxide has been shown to create virus inactivation, at least in influenza strains [57]. Titanium oxide nanoparticles have been shown to be non-poisonous [58] in some studies and cytotoxic in others [59].

## Zinc

While zinc oxide nanoparticles have been shown to be cytotoxic, [58] there are antibacterial benefits [60]. Specifically, zinc oxide is an effective, and promising antiviral agent against the H1N1 influenza virus [61]. Due to a variety of mechanisms, zinc has been suggested as an adjunct for treatment for COVID-19 respiratory infections, [62] mostly due to the observed effect zinc ions have on the RNA polymerase of the corona virus [63]. At the same time, it has been shown that reversible airway inflammation can occur after inhalation of zinc oxide nanoparticles [64].

## Magnesium

Magnesium oxide is usually less expensive than the majority of other metallic ion nanoparticles. Magnesium oxide and its nanoparticles have shown antimicrobial activity [65] but studies have shown that it is necessary to identify the safe critical concentration of Mg and polymer, which prevents bacterial infections [66]. Mazaheri et.al. suggested that magnesium oxide nanoparticles in concentrations lower than 250 μg.mL^− 1^ are safe for desired applications [67]. In food borne bacterial infections, magnesium has had tremendous success as a nanoparticle [68]. Combining zinc and magnesium oxide nanoparticles has shown additive effects in relation to specific bacterial infections, and the fact that they are inexpensive, available, and biocompatible makes them an attractive option [69, 70]. The viricidal and antiviral activity of magnesium oxide nanoparticles has been shown with in vitro foot and mouth disease [71], and magnesium oxide has been suggested as a potential virucide with herpes simplex virus type 1 (HSV-1) [72].

## Copper

The attraction of combining copper and PLA to print these replacement parts is easy to see. The commercially available copper/PLA market is readily accessible. There is evidence that copper can help reduce the risk of influenza virus environmental contamination when impregnated within masks [73]. Additionally, copper has been seen to have antibacterial and antiviral potential especially when in the presence of an oxidizing agent [74, 75]. When comparing the viability of COVID-19 on plastic versus copper, [76] a potential advantage to combining these two materials has not been examined, but is plausible. While the microbiological effects of copper are positive, there are potential cytotoxic issues [77, 78]. In fact, copper nanoparticles are shown to potentially have the most cytotoxic effects [79] compared to other ionic nanoparticles.

## Silver

Silver nanoparticles have broad antimicrobial activities specifically showing activity against *Escherichia coli* and *Staphylococcus aureus* [80]. As far as viral effectiveness, silver nanoparticles has been shown effective against both human immunodeficiency virus (HIV) [81] the respiratory syncytial virus (RSV) [82] and adenovirus, [83] but not in the context of FDM or printing PPE. While there does exist questions of its safety, recently a limit of 0.19 *μ* g/m^3^ for silver nanoparticles has been suggested based on a rat-inhalation toxicity study [84].

## Conclusion

While van Doremalen et.al. noted the viability of severe acute respiratory syndrome coronavirus 2 (SARS-CoV-2) on plastic, [76] PP was the only plastic evaluated. Although PLA has been evaluated in areas of material strength and effects after sterilization, [85, 86] more needs to be done to evaluate the use of these materials as potential replacement parts in PAPRs. Nanoparticles combined with PLA or other polymers are promising options for printing replacement parts because of their biological properties as antimicrobials. However, it must be remembered that they can possibly lead to adverse biological effects at the cellular levels. The toxicity of nanoparticles can vary depending on their size, morphology, surface area, surface reactivity, and solubility [87] and this means that future research should balance the safety with the effectiveness of 3D printed materials. The current three examples of utilizing CT scanning, segmentation, and additive manufacturing, to produce desperately needed replacement parts for compromised PAPRs is only the beginning of the possibilities that have been foreshadowed. While a significant amount of research should still be done, this may serve as an example of how to create a stop gap with our current technology to help us flatten the curve and protect those on the frontline of COVID-19.

## Data Availability

Data sharing is not applicable to this article as no datasets were generated or analyzed during the current study.

## Abbreviations

CT: Computed tomography
MRI: Magnetic resonance imaging
PET: Positron emission tomography
PPE: Personal protective equipment
PAPR: Powered air-purifying respirator
CDC: Centers for Disease Control and Prevention
CHI: Catholic Health Initiatives
DICOM: Digital imaging and communications in medicine
PLA: Polylactic acid
STL: Stereolithography
WHO: World health organization
IP: Intellectual property
PAHPRA: Pandemic and All-Hazards Preparedness Reauthorization Act
FDA: Food and Drug Administration
DHHS: Department of Health and Human Services
HSV-1: Herpes simplex virus type 1
ASTM: American Society for Testing and Materials
FDM: Fused deposition modeling
EUA: Emergency Use Authorization
TPE: Thermoplastic elastomer
TPU: Thermoplastic polyurethane
PCL: Polycaprolactone
PET: Polyethylene terephthalate
PETG: Polyethylene terephthalate glycol-modified
PETT: Polyethylene cotrimethylene terephthalate
PEEK: Polyether ether ketone
ABS: Acrylonitrile butadiene styrene
PC: Polycarbonate
PVA: Polyvinyl alcohol
HIPS: High impact polystyrene
POM: Polyoxymethylene
PMMA: Polymethyl methacrylate
FPE: Flexible polyester
HDPE: High-density polyethylene
TPC: Thermoplastic copolyester
ASA: Acrylonitrile styrene acrylate
PP: Polypropylene
PPSU: Polyphenylsulfone
HIV: Human immunodeficiency virus
RSV: Respiratory syncytial virus
SARS-CoV-2: Severe acute respiratory syndrome coronavirus type 2
